# NR2F2 Regulates Cell Proliferation and Immunomodulation in Whartons’ Jelly Stem Cells

**DOI:** 10.3390/genes13081458

**Published:** 2022-08-16

**Authors:** Li Ma, Min Huang, Xiaohua Liao, Xiyu Cai, Qiang Wu

**Affiliations:** 1 The State Key Laboratory of Quality Research in Chinese Medicine, Macau University of Science and Technology, Macau 999087, China; 2 Department of Orthopedic, The Fifth Affiliated Hospital of Sun Yat-sen University, Zhuhai 519000, China

**Keywords:** WJ-MSCs, NR2F2, RNA-seq, gene regulatory network, cell cycle, immunomodulation

## Abstract

(1) Background: Wharton’s Jelly stem cells (WJ-MSCs) are multipotent mesenchymal stem cells that can proliferate rapidly and have low immunogenicity. Therefore, WJ-MSCs have gained considerable attention in the fields of immunomodulation and disease treatment and have entered clinical trials for the treatment of various diseases. Therefore, it is crucial to study the underlying mechanisms of WJ-MSCs proliferation, immune regulation, and disease treatment. Nuclear Receptor Subfamily 2 Group F Member 2 (NR2F2) is a transcription factor that is involved in the regulation of many different genes. However, it remains unknown how NR2F2 regulates stem cell identity in WJ-MSCs. (2) Methods: We used RNAi technology to knock down NR2F2 in WJ-MSCs, and studied the regulatory role of NR2F2 in WJ-MSCs by MTT, flow cytometry, RNA-seq, and other methods. We also utilized a co-culture system in which NR2F2-depleted WJ-MSCs with MH7A and HCT116/HepG2 were used to investigate the role of NR2F2 in immunomodulation and the inhibition of cancer cell growth. (3) Results: NR2F2 knockdown resulted in decreased expressions of Cyclin D1 and CDK4, slower cell proliferation, and increased expressions of IL6 and IL8. Furthermore, Cyclin D1, CDK4, and inflammatory factors were increased in human rheumatoid fibroblast-like synoviocyte line MH7A if co-cultured with NR2F2 depleted WJ-MSCs. In addition, we observed increased p53, decreased BCL-2, and increased cell apoptosis in liver cancer cell line HepG2 if co-cultured with NR2F2-depleted WJ-MSCs. (4) Conclusions: NR2F2 not only plays an important role in the cell cycle and immune regulation of WJ-MSCs but also has potential effects on the WJ-MSCs treatment of related diseases.

## 1. Introduction

Wharton’s Jelly mesenchymal stem cells (WJ-MSCs) are derived from the mucin-like connective tissue in the umbilical cord. WJ-MSCs highly express mesenchymal stem cell (MSC) surface markers CD150 and CD44 and have a low expression of CD106. They neither express hematopoietic cell markers CD14, CD34, CD45, nor endothelial marker CD31. Interestingly, compared with typical mesenchymal stem cells, WJ-MSCs retain some pluripotency markers OCT4, SOX2, and NANOG [1,2]. Therefore, WJ-MSCs have a wider range of differentiation and can be induced to differentiate into many cell types, including osteocytes and chondrocytes, adipocytes, nerve cells, cardiomyocytes, muscle cells, epidermal cells, endothelial cells, retinal progenitor cells, insulin-producing cells, and hepatocytes. WJ-MSCs are in a more primitive state and thus less immunogenic. In addition, WJ-MSCs are highly proliferative while holding a wide spectrum of differentiation potential. Since they are easy to isolate and store, WJ-MSCs provide a safe stem cell source for stem cell therapy and tissue engineering. Indeed, WJ-MSCs have been used for liver injury, kidney injury, skin injury, spinal cord injury, chronic graft-versus-host disease, premature ovarian failure, Parkinson’s disease, Alzheimer’s disease, and other diseases and achieved good treatment effects [3,4,5,6]. Hence, WJ-MSCs have important clinical value and broad application prospects.

Compared with human embryonic stem cells (hESCs) and other mesenchymal stem cells, WJ-MSCs have unique transcriptomic profiles. For example, compared with BM-MSCs, WJ-MSCs have higher undifferentiated hESC markers such as NANOG, DNMT3B, and GABRB3, pluripotent stem cell markers, and some early endoderm markers at early and late stages [7]. WJ-MSCs possess the properties of true stem cells and can be retained even after prolonged in vitro culture. Stem cell-related markers such as IL6ST, PTEN, and COMMD3 are also expressed in WJ-MSCs [8]. In addition, compared with other types of stem cells, the most variable and up-regulated genes in WJ-MSCs are IL8, SRGN, IL33, PTGS2, CXCL1, SEMA3C, etc. These genes are mostly related to immunity, chemotaxis, and cell death [9].

NR2F2 is a member of the chicken ovalbumin upstream promoter-transcription factor (COUP-TF) family. Its sequence is highly conserved and expressed in the mesenchymal tissues of many organs that require mesenchymal–epithelial interaction [10,11], and it plays an important role in regulating the cell cycle, maintaining cell survival, and regulating cell differentiation [10,11,12]. NR2F2 can regulate tumor growth and metastasis by regulating tumor angiogenesis [13]. In addition to its role in cancer, there are also related studies on the stemness regulatory function of NR2F2. Studies have shown that NR2F2 is highly expressed in differentiated cells and adult mesenchymal cells, and its expression affects terminal cell differentiation [14]. The inhibition of NR2F2 impairs the self-renewal and pluripotency of bone marrow mesenchymal stem cells (BM-MSCs), indicating that NR2F2 plays an important role in BM-MSCs [15]. However, whether NR2F2 affects the proliferation and immunoregulatory properties of WJ-MSCs has not been reported.

As a promising type of MSCs, WJ-MSCs have not yet been clearly reported on their biological properties and molecular biological mechanisms regulating immunotherapy diseases. In order to gain an in-depth understanding of the role of WJ-MSCs in the treatment of related diseases and the effect of NR2F2 on them, we aimed to investigate the regulatory role of NR2F2 in multipotency maintenance, cell proliferation, and the immunomodulation of WJ-MSCs. Our results can provide novel insights into gene regulation in WJ-MSCs and provide new cues for their applications.

## 2. Materials and Methods

### 2.1. Cell Culture

WJ-MSCs cells obtained from Cyagen were cultured in DMEM/High Glucose (Gibco™, Thermo Fisher, Waltham, MA, USA) supplemented with 20% fetal bovine serum (Gibco™, Thermo Fisher, Waltham, MA, USA). MH7A, HepG2, and HCT116 obtained from ATCC were maintained in DMEM/High Glucose supplemented with 10% fetal bovine serum. All of the cells were incubated at 37 °C in a 5% CO_2_ incubator. The medium was changed daily, and the cells were passaged every 2 to 3 days.

### 2.2. Immunofluorescence Staining

The cells cultured in 6-well dishes were fixed in 4% paraformaldehyde and permeabilized with 0.25% Triton X-100, followed by blocking with 3% BSA in PBS. Then cells were probed with the primary antibody in 3% BSA for 1 h at 4 °C and the secondary antibody in 3% BSA for 30 min at room temperature. A drop of Vectashield mounting medium with 49, 6-diamidino-2-phenylindole (DAPI; C1006, Beyotime, Shanghai, China) was placed on the microscope slide, and the coverslip was sealed with nail polish in a way that the cells were in contact with the mounting medium. The staining signal was then observed through the microscope. The primary antibodies used were: anti-NR2F2 (ab211777, Abcam, Cambridge, UK), and anti-Ki67 (sc-23900, Santa Cruz, Dallas, TX, USA).

### 2.3. RNA Interference (siRNA)

The WJ-MSCs were placed in a 6-well plate. NR2F2 siRNA (sc-38818, Santa Cruz) and the scrambled control siRNA (sc-37007, Santa Cruz). Reverse transfections with siRNA were performed with LipoRNAi™ Transfection Reagent according to the manufacturer’s instructions. The cells were maintained in a 20% FBS-containing medium for the duration of the experiment for 72 h.

### 2.4. Real-Time Quantitative PCR (qPCR)

The total RNA was isolated by Trizol reagent according to the manufacturer’s instructions. The cDNA was synthesized from the total RNA using the Superscript III First-Strand Synthesis System with an oligo-dT primer (Invitrogen, Waltham, MA, USA). Quantitative real-time PCR was performed using Fast SYBR Green Master Mix (Bio-Rad Laboratories, Hercules, CA, USA). The PCR reactions were performed at 95 °C for 10 min, followed by 40 cycles of 95 °C for 15s, 60 °C for 60 s, and 72 °C for 1 min. The relative quantification of mRNA levels was computed using the 2^−^^△△Ct^ method. β-actin served as the internal reference gene. The primers used for RT-qPCR analysis are shown in Table 1.

### 2.5. Western Blot

Firstly, the cells were washed twice with PBS prior to sample preparation. RIPA lysis solution (P0013C, Beyotime, Shanghai, China) was used to lyse the cells at 4 °C for 30 min. A BCA kit (P0010S, Beyotime, Shanghai, China) was used to detect the total protein concentration. Then, SDS–PAGE loading buffer (P0015F, Beyotime, Shanghai, China) was added to the lysate, and the mixture was boiled at 100 °C for 5 min. Then, 10–20 µg of protein per sample was separated with SDS-PAGE gels. Next, the proteins were transferred onto polyvinylidene fluoride (PVDF) membranes (Millipore, Billerica, MA, USA, ISEQ00010). The blots were incubated overnight at 4 °C with the corresponding primary antibodies and incubated with secondary antibodies at room temperature for 1 h, and developed with ECL substrate solutions A and B (Pierce™, Thermo Fischer). The primary antibodies used were: mouse anti-CDK4(sc-23896, Santa Cruz), mouse anti-Cyclin D1(sc-8396, Santa Cruz), mouse anti-p53(sc-126, Santa Cruz), mouse anti-BCL-2(sc-7382, Santa Cruz), mouse anti-BAX (sc-7480, Santa Cruz), mouse anti-NF-kB (sc-8008, Santa Cruz), mouse anti-Caspase3(sc-7272, Santa Cruz), and mouse anti-β-actin (sc-47778, Santa Cruz).

### 2.6. RNA-Seq

Before the RNA was collected, the NR2F2 in WJ-MSCs were knocked-down. When the RNA was collected, the cells were 80% confluent and healthy-looking. RNA-seq library preparation and 100 bp pair-end sequencing on the DNBSEQ platform. FastQC checks were performed on the raw data. RNA-seq reads were aligned to the human genome (hg19 build) and checked for differential expression. Downstream analysis was performed as described in the text.

### 2.7. MTT Assay

The cells were placed in a 24-well plate that had been pre-incubated for 24 h. WJ-MSCs were transfected with NR2F2 siRNA or control siRNA for 72 h. The transfected cells were maintained in the medium. Then, 1% MTT was added. The plates were incubated for 4 h at 37 °C. Formazan was dissolved with DMSO. Absorbances were measured at 490 nm using a microplate reader.

### 2.8. Cell Cycle and Apoptotic Analysis

Cell cycle analysis was measured by a cell cycle and apoptosis analysis kit (FXP0211, 4A BIOTECH, Beijing, China). Briefly, the cells were fixed and stained with PI. Flow cytometry was performed using the BD Flow Cytometry Analyser. The apoptotic activity in cells was measured by an Annexin V-FITC/PI apoptosis detection kit (FXP018, 4A BIOTECH, Beijing, China), according to the manufacturer’s instructions. Briefly, the cells were washed with pre-cooled PBS and resuspended in Annexin V binding solution. Next, the cell suspension was incubated with Annexin V-FITC in the dark and then with Propidium Iodide. The cells were analyzed by flow cytometry.

### 2.9. Statistical Analysis

All of the experiments were conducted in triplicate. A student’s *t*-test was applied for statistical analysis and the results with a mean of ±SE. *p* < 0.05 was considered significant (* *p* < 0.05, ** *p* < 0.01, *** *p* < 0.001)

## 3. Results

### 3.1. NR2F2 Depletion Affects Genes Associated with Stem Cell Identity and Immune Response in WJ-MSCs

**Figure 1:**
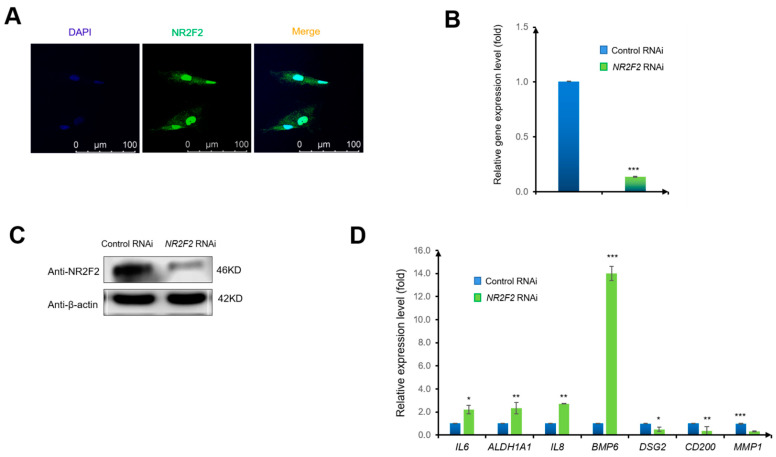
*NR2F2* knockdown affects key factors of immunity and differentiation in WJ-MSCs. (**A**) Immunocytochemical staining for DAPI (blue) and NR2F2 (green). Images were taken at 40× magnification. Scale bar = 100 μM. (**B**) NR2F2 mRNA levels (relative to β-actin) in WJ-MSCs transfected with control siRNA (control siRNA), or NR2F2 siRNA (NR2F2 RNAi) were analyzed by qPCR. The data were analyzed by the δ-δ-Ct method, and the genes in control siRNA-transfected WJ-MSCs were set to 1. (**C**) Western blot analysis of NR2F2 protein levels in WJ-MSCs. (**D**) Relative gene expression changes of *IL6*, *ALDH1A1*, *IL8*, *BMP6*, *DSG2*, *CD200*, and *MMP1* genes upon NR2F2 depletion (control RNAi as control). * *p* ≤ 0.05, ** *p* ≤ 0.01, *** *p* ≤ 0.001.

To examine the gene expression pattern of NR2F2 in WJ-MSCs, we used an immunofluorescence technique with an anti-NR2F2 antibody. We observed that NR2F2 was mainly expressed in the nucleus of WJ-MSCs (Figure 1A). Next, to explore the function of NR2F2, we knocked down NR2F2 by transfecting NR2F2 siRNA into WJ-MSCs (control siRNA as negative control). We observed that the RNA level of NR2F2 was drastically reduced to 13% compared with the control RNAi after 72 h RNAi treatment (Figure 1B). Consistently, the protein level of NR2F2 was also substantially decreased (Figure 1C). Interestingly, NR2F2 depletion led to significant changes in the expression levels of several important genes related to immunity and differentiation in WJ-MSCs. The immune-related genes *IL6*, *ALDH1A1*, *IL8*, and *BMP6*, were up-regulated to 2.22 fold, 2.34 fold, 2.72 fold, and 14.01 fold, respectively. In contrast, the MSC-related genes *DSG2*, *CD200*, and *MMP1* were reduced to a down-regulation of 0.50 fold, 0.38 fold, and 0.32 fold, respectively (Figure 1D). These results indicated that NR2F2 might have an important regulatory role in WJ-MSCs.

### 3.2. NR2F2 Regulates Genes Which Are Important for Cell Growth and Immunity in WJ-MSCs

**Figure 2:**
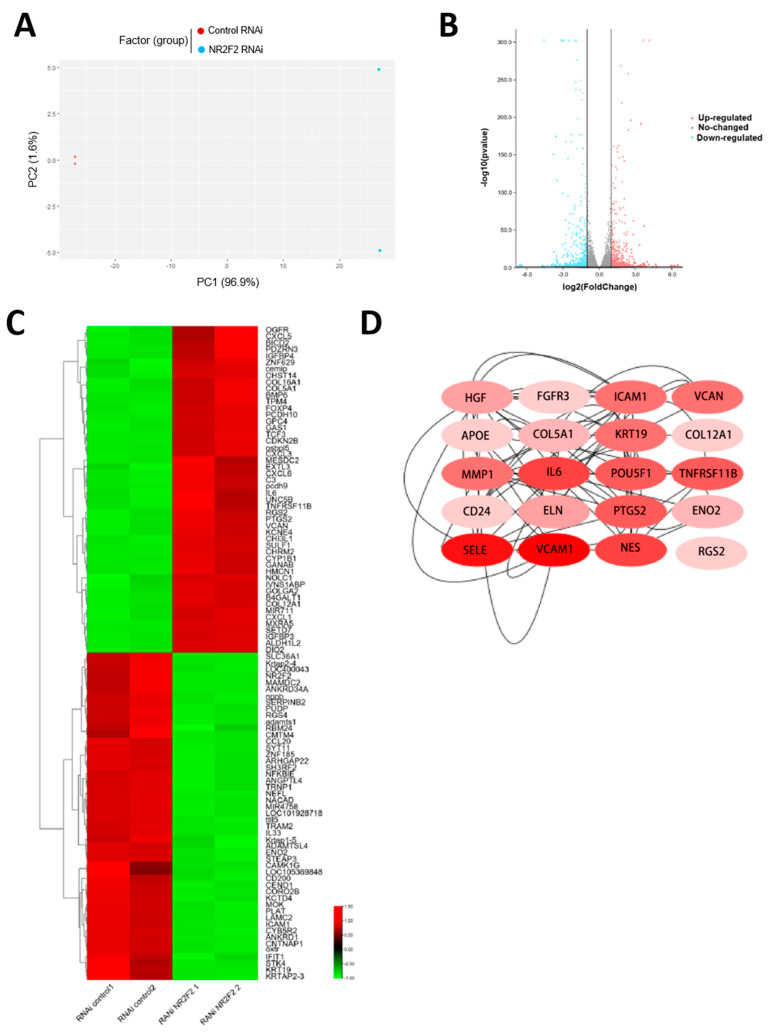
Global gene expression changes upon NR2F2 knockdown in WJ-MSCs. (**A**) WJ-MSCs exhibited two distinct gene expression patterns after NR2F2 was knocked down. (**B**) Each point in the volcano plot represents a gene. Red dots indicate significantly up-regulated genes, blue dots indicate significantly down-regulated genes, and grey dots indicate non-differentially expressed genes. (**C**) Heatmap showing the top 50 genes with the most significant up- and down-regulation, with red for up-regulation and green for down-regulation. (**D**) The darker the red of the Hub gene, the higher the score.

To further characterize the role of NR2F2 in WJ-MSCs, we performed RNA sequencing on WJ-MSCs treated with the knockdown of NR2F2. Principal component analysis showed that the samples were clearly clustered into two distinct groups based on the NR2F2 knockdown treatment (Figure 2A). After the down-regulation of NR2F2 gene expression, 514 genes were up-regulated (log2FoldChange > 1, padj < 0.05) and 495 genes were down-regulated (log2FoldChange < −1, padj < 0.05) (Figure 2B). Notably, most of the top 100 most altered genes were associated with cell growth and immune regulation (Figure 2C). After identifying the differential genes, the cytoHubba plugin in Cytoscape was used to find the top 20 key genes in the differential genes, and the highest score was IL6 (Figure 2D). These genes are related to immunity, differentiation, and cell growth.

**Figure 3:**
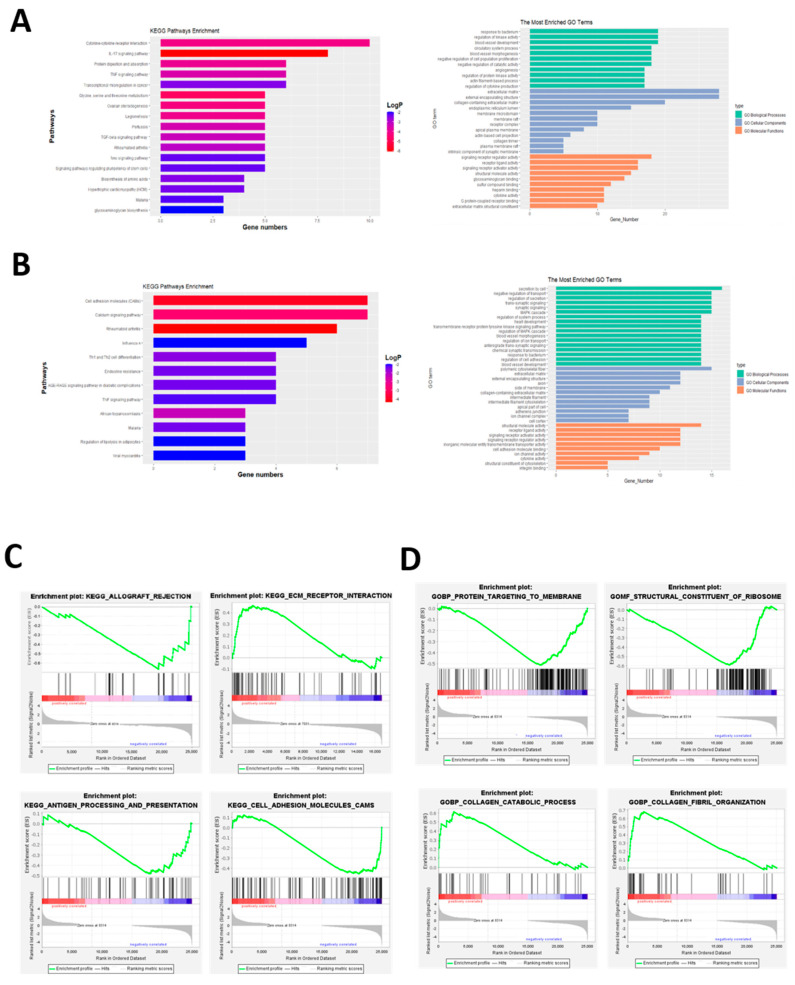
RNA-seq differential gene enrichment analysis identified pathways significantly altered by NR2F2. (**A**) KEGG and GO enrichment pathways of up-regulated genes. (**B**) KEGG and GO enrichment pathways of down-regulated genes. (**C**) KEGG analysis of GSEA. (**D**) GO analysis of GSEA.

NR2F2 is a nuclear receptor family member and a transcription factor, and we observed the significant global gene expression changes upon NR2F2 depletion. To better understand how NR2F2 mediates its diverse functions in WJ-MSCs, we next aimed to explore signaling pathways that are associated with NR2F2. To achieve this, we performed KEGG and GO enrichment analysis using up-regulated genes and down-regulated genes, respectively. The up-regulated pathways mainly involved cytokine receptor signaling pathway, IL17 signaling pathway, TNF signaling pathway, extracellular matrix, and other related pathways, stem cells pluripotency and other signaling pathways (Figure 3A), down-regulated pathways mainly involve cell adhesion molecules, calcium signaling pathways, and rheumatoid arthritis (Figure 3B). To further identify enriched pathways, we performed Gene Set Enrichment Analysis (GSEA) using the p-values associated with each gene and the pre-sorted gene list analysis option. We found that the enrichment results were associated with cell adhesion and the cell matrix (Figure 3C,D).

### 3.3. NR2F2 Affects the Biological Properties of WJ-MSCs

**Figure 4:**
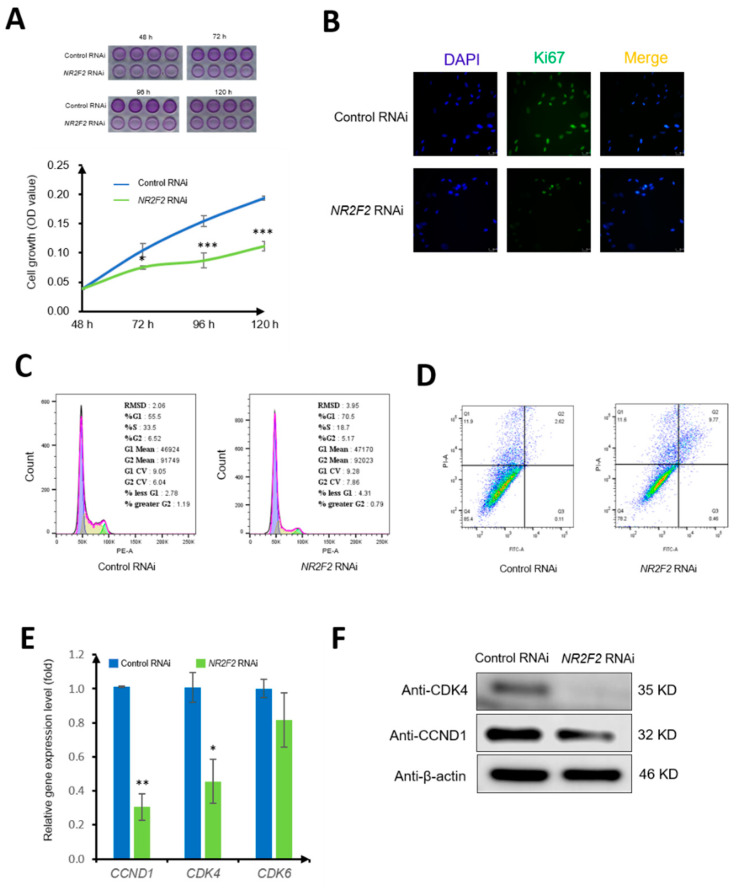
NR2F2 regulates WJ-MSC proliferation and cell cycle. (**A**) Growth curves of WJ-MSCs under control RNAi and *NR2F2* RNAi conditions. Each time point in each row is n = 4. (**B**) Immunocytochemical staining for DAPI (blue) and Ki67 (green). (**C**) Flow cytometry to examine cell cycle changes. (**D**) Cell apoptosis was detected by flow cytometry. (**E**) qPCR results, relative expression levels of *CCND1*, *CDK4*, and *CDK6* genes in control (control RNAi) and NR2F2 knockdown (*NR2F2* RNAi) groups. (**F**) Western Blot analysis of cyclin CCND1, CDK4 expression. * *p* ≤ 0.05, ** *p* ≤ 0.01, *** *p* ≤ 0.001.

To affirm the regulatory role of NR2F2 in the cell proliferation of WJ-MSCs. We used an MTT assay to observe the living cells at 48 h, 72 h, 96 h, and 120 h after transduction. From 72 h, the growth rate of cells in the NR2F2 RNAi group was lower than that in the control RNAi group (*p* < 0.05) (Figure 4A). Immunofluorescence staining was used to observe the proliferation marker Ki67, and the fluorescence intensity of the NR2F2 RNAi group was lower than that of the control RNAi group (Figure 4B). in addition, our cell cycle analysis showed that 55.5% of the cells were in the G1 phase, 33.5% were in the S phase, and 6.52% were in the G2 phase in the control RNAi group. In contrast, the cells in the G1 phase accounted for 70.5%, the S phase accounted for 18.7%, and the G2 phase accounted for 5.17% upon NR2F2 RNAi, indicating that the cells in the G1 phase were increased by 15% after NR2F2 knockdown (Figure 4C). Furthermore, our apoptotic cell results demonstrated that the early apoptotic cells accounted for 0.11%, and the late apoptotic cells accounted for 2.62% in the control RNAi group. In the NR2F2 RNAi group, apoptosis was increased by 7.5% after NR2F2 knockdown (Figure 4D). Taken together, our results indicated that the cell cycle was blocked, and apoptosis increased after NR2F2 knockdown, suggesting that NR2F2 is important in regulating cell proliferation. However, further studies will be needed to determine whether NR2F2 can directly regulate cell proliferation.

In order to further clarify how NR2F2 regulates cell cycle changes, we used qPCR to detect the changes of *CCND1*, *CDK4*, and *CDK6* cell cycle-related genes. The results showed that the expression of *CCND1* and *CDK4* decreased after NR2F2 knockdown (*p* < 0.05). The Western Blot results also showed that the Cyclin protein expressions of D1 and CDK4 were decreased, which was consistent with mRNA expression (Figure 4E). NR2F2 may affect cell growth by regulating the expression of *CCND1* and *CKD4*.

### 3.4. NR2F2-Depleted-WJ-MSCs Affect the Growth of MH7A and the Secretion of Inflammatory Factors

**Figure 5:**
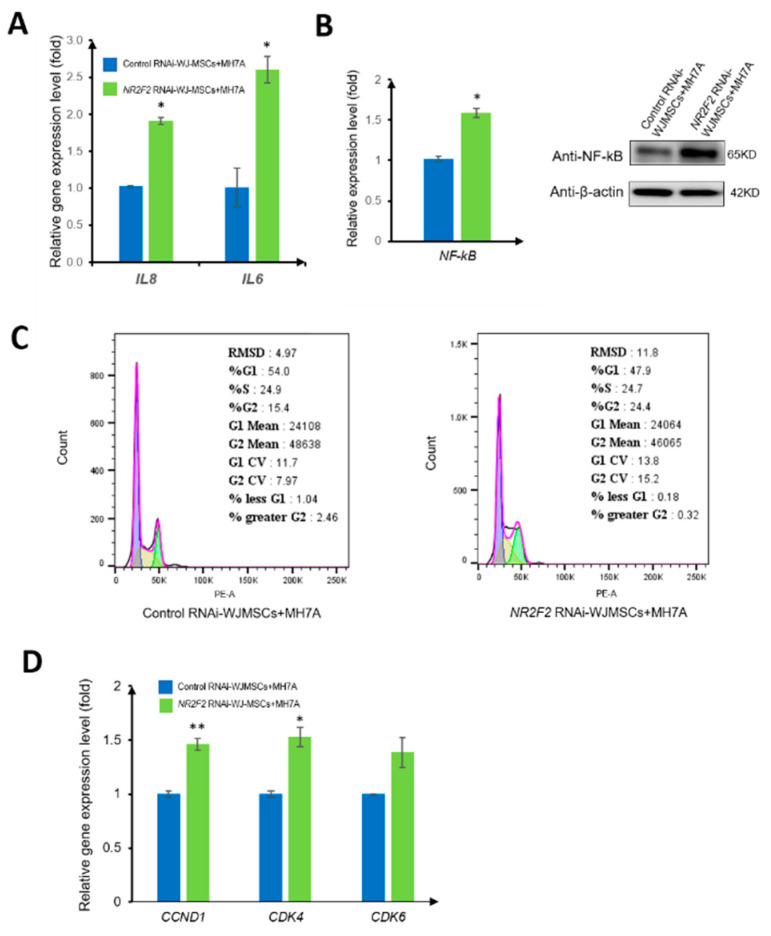
NR2F2 affects the co-culture of WJ-MSCs with MH7A. (**A**) The relative expression levels of *IL8* and *IL6* in MH7A co-cultured with control RNAi and *NR2F2* RNAi-treated WJ-MSCs were detected by qPCR. (**B**) mRNA expression and protein expression of *NF*-*kB* in different treatment groups. (**C**) Flow cytometry to examine cell cycle changes. (**D**) qPCR results, relative expression levels of *CCND1*, *CDK4*, and *CDK6* genes. * *p* ≤ 0.05, ** *p* ≤ 0.01.

To probe whether WJ-MSCs have an immunomodulation ability, we co-cultured NR2F2 depleted WJ-MSCs with MH7A (a typical rheumatoid fibroblast-like synoviocyte cell line) for 72 h; the important inflammatory factors IL-6 and IL-8 in MH7A were detected, and the expressions of both increased (Figure 5A). The mRNA and protein expressions of NF-kB, which play an important role in the study, showed that the expression of NF-kB was increased (Figure 5B). The increased secretion of inflammatory factors may be related to the NF-kB pathway. The NF-kB signaling pathway not only regulates immune inflammation but also affects cell proliferation. Therefore, we examined the cell cycle changes of MH7A co-cultured with WJ-MSCs under different treatment conditions. We found that the MH7A cells co-cultured with the control RNAi group accounted for 54.0% in the G1 phase, 24.9% in the S phase, and 15.4% in the G2 phase. Strikingly, MH7A cells co-cultured with NR2F2-knockdown WJ-MSCs accounted for 47.9% in the G1 phase, 24.7% in the S phase, and 24.4% in the G2 phase. The proportion of the G2 phase increased by 9%, and the ratio of the G2 phase of MH7A was increased (Figure 5C). Further, the mRNA expression levels of *CCND1*, *CDK4*, and *CDK6* in MH7A were increased (Figure 5D), suggesting that cell proliferation was accelerated.

### 3.5. NR2F2-Depleted WJ-MSCs Have Different Effects on Different Cancer Cells

**Figure 6:**
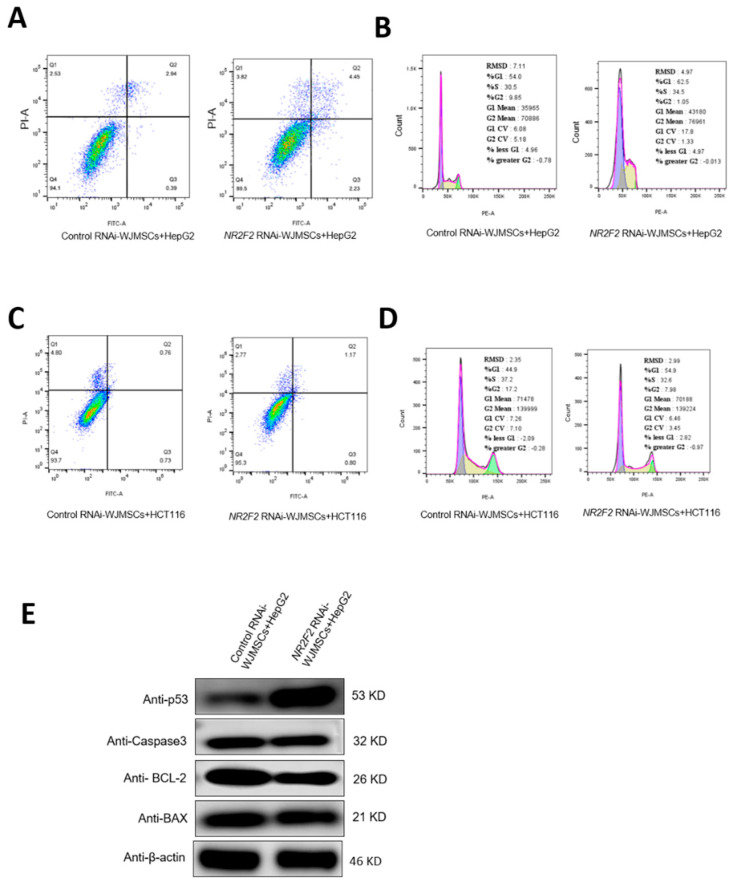
NR2F2-depleted WJ-MSCs regulate the growth of HepG2 and HCT116 cells in different ways. (**A**) Apoptosis of HepG2 cells. (**B**) HepG2 cell cycle changes. (**C**) Apoptosis of HCT116 cells. (**D**) HCT116 cell cycle changes. (**E**) Western blot analysis of apoptosis-related protein expression.

Studies have shown that WJ-MSCs can inhibit the growth of cancer cells since we observed that NR2F2 in WJ-MSCs can affect various signaling pathways such as intercellular adhesion, cell chemokines, and cytokine receptors. Importantly, we speculated that NR2F2 might also play a role in the inhibition of cancer cell growth by WJ-MSCs. To test this conjecture, we co-cultured WJ-MSCs in the Control RNAi group and WJ-MSCs in the NR2F2 RNAi group with HepG2 and HCT116 cells, respectively, to observe whether NR2F2 played a role in the inhibition of cancer by WJ-MSCs. Our results showed that the HepG2 cells co-cultured with the Control RNAi group accounted for 0.39% of the early apoptotic cells and 2.94% of the late apoptotic cells. HepG2 cells co-cultured with the NR2F2 RNAi group accounted for 2.23% of early apoptotic cells and 4.45% of late apoptotic cells, with increased apoptosis (Figure 6A). The results of cell cycle detection showed that the HepG2 cells co-cultured with the Control RNAi group accounted for 54.0% in the G1 phase, 30.5% in the S phase, and 9.85% in the G2 phase. The HepG2 cells co-cultured with WJ-MSCs in the NR2F2 RNAi group accounted for 62.5% in the G1 phase, 34.5% in the S phase, and 1.05% in the G2 phase. The proportion of HepG2 cells co-cultured with NR2F2-knockdown WJ-MSCs in the G2 phase was reduced by 8.8% (Figure 6B).

We also tested the results of the co-culture with HCT116. We found that the early apoptotic cells accounted for 0.73%, and late apoptotic cells accounted for 0.76% in the Control RNAi group. In the *NR2F2* RNAi group, early apoptotic cells accounted for 0.8%, and late apoptotic cells accounted for 1.17%. NR2F2 knockdown had no obvious effect on HCT116 cell apoptosis (Figure 6C). The cell cycle results showed that the HCT116 cells co-cultured with the control RNAi group accounted for 44.9% in the G1 phase, 37.2% in the S phase, and 17.2% in the G2 phase. The HCT116 cells co-cultured with NR2F2 knockdown WJ-MSCs accounted for 54.9% in the G1 phase, 32.6% in the S phase, and 7.98% in the G2 phase. The proportion of HCT116 cells co-cultured with NR2F2 knockdown WJ-MSCs increased by 10% in the G1 phase and decreased by 9.22% in the G2 phase. The proportion of the S phase + G2 phase in the cell cycle decreased by 13.82%. These suggested that NR2F2-depleted WJ-MSCs slowed down the proliferation of HCT116 cells (Figure 6D).

To further verify the results of apoptosis in the HepG2 cells after co-culturing, we used Western Blot to detect the expression of apoptosis-related proteins in the HepG2 cells. Indeed, we observed increased p53 and decreased BCL-2 (Figure 6E), which was consistent with the results of flow cytometry; this confirmed that NR2F2-depleted WJ-MSCs increased HepG2 apoptosis.

## 4. Discussion

We proposed that NR2F2 is an important transcription factor in WJ-MSCs. Previous studies have demonstrated that NR2F2 can increase the expression of Cyclin D1 and p21 [16], and the deletion of NR2F2 results in reduced proliferation [17]. NR2F2 can directly activate enhancer elements on both sides of cell cycle genes to drive their expression [18]. Further, NR2F2 can bind to the E2F1 promoter and directly mediate the expression of E2F1 to regulate the cell cycle [19]. Therefore, one of our aims of this study was to explore the function of NR2F2 in regulating the cell proliferation of WJ-MSCs. To this end, we knocked down *NR2F2* in WJ-MSCs to observe its effect. Our findings suggest that NR2F2 plays a role in immune regulation and cell proliferation in WJ-MSCs. In WJ-MSCs, NR2F2 can regulate the cell cycle by affecting the expression of Cyclin D1 and CDK4 and affect cell proliferation.

WJ-MSCs possess immunomodulatory properties, making them a good option for the treatment of autoimmune diseases. WJ-MSCs can exert immunomodulatory effects by secreting soluble factors [20]. In general, WJ-MSC therapy can effectively control the progression of autoimmune diseases. WJ-MSCs produce large amounts of resistant interleukin 10 (IL-10), transforming growth factor-β (TGF-β) [21,22,23], and it also expresses IL-6, vascular endothelial growth factor (VEGF), and CD200, which is very important for the immunosuppressive ability of MSCs [21,24,25]. Our next aim is to study whether NR2F2 plays an immunomodulatory role in WJ-MSCs. In our study, we demonstrated that NR2F2 could also regulate the expression of IL-6, IL-8, and CD200, highlighting that NR2F2 has an impact on the immunomodulatory properties of WJ-MSCs.

Rheumatoid arthritis is a chronic autoimmune disease that has been treated with MSCs in clinical studies. IL-6, IL-8, and CD200 play an important role in arthritis and other autoimmune diseases [26,27]. MSCs can reduce the level of pro-inflammatory cytokines, inhibit joint swelling and cartilage erosion, and then treat rheumatoid arthritis [28]. It suppresses the activation, migration, and invasion of rheumatoid arthritis fibroblast-like synovial cells by increasing the expression of miR-320a in exosomes secreted by mesenchymal stem cells and attenuates arthritis and bone damage [29]. Treating rheumatoid arthritis with WJ-MSCs not only improves symptoms but has the potential to inhibit the progression of the disease. Our third aim is to investigate whether NR2F2 can inhibit inflammation when NR2F2 depleted WJ-MSCs co-cultured with MH7A. We found that the expressions of important pro-inflammatory cytokines IL-6, IL-8, and NF-kB in MH7A cells were increased when NR2F2 was depleted. Hence, NR2F2 may have implications for rheumatoid arthritis therapy.

Most studies have shown that stem cells can inhibit the proliferation of cancer cells, but some in vitro experiments have shown that stem cells can promote the proliferation of cancer cells [30]. The reason why some studies suggest that stem cells promote the proliferation of cancer cells may be related to the type of cancer cells. Although there are relatively few studies on the anti-cancer properties of WJ-MSCs, all of the currently available studies suggest that WJ-MSCs can inhibit the proliferation of cancer cells and have stronger anti-cancer properties than MSCs from other sources [31]. Therefore, WJ-MSCs are a potential cancer therapy. Compared with other mesenchymal stem cells, the most highly expressed oncogene and tumor suppressor genes in WJ-MSCs are PDGFRA and TGFBR2 [4], which may be related to their strong anti-cancer properties. Our fourth aim was to probe the possible anti-cancer properties of NR2F2. We used NR2F2 depleted WJ-MSCs co-cultured with HepG2 and HCT116 cells and found that NR2F2 depleted WJ-MSCs can affect the proliferation and apoptosis of cancer cells. Based on our RNA-seq data, we speculate that the underlying mechanism could be that NR2F2 can regulate some cancer-related genes, including *PDGFRA*, *TGFBR2*, *p53*, and *BCL-2*. As in our study, the RNA-seq results showed that PDGFRA was down-regulated, TGFBR2 was up-regulated, and the expression of related apoptosis markers was increased after NR2F2 knockdown, suggesting that NR2F2 promotes the effect of WJ-MSCs in inhibiting cancer cell proliferation. Additionally, MSCs can interact with cytokines such as IL-8, transforming growth factor-ss1 (TGF-ss1) and neurotrophic factor-3, thereby exerting its anti-cancer effect [32].

Previous studies have shown that NR2F2 expression was positively correlated with cell invasion, migration, and the expression of N-cadherin and vimentin [33] and has a key role in ERα-mediated transcription [34]. NR2F2 can also promote tumor cell proliferation, epithelial-mesenchymal transition, and invasive characteristics and inhibit tumor differentiation and immune cell infiltration by regulating transcriptional programs commonly found in mouse and human squamous cell carcinomas [35]. The downregulation of NR2F2 inhibits cancer cell proliferation and EMT [36]. Hence, NR2F2 may play a direct role in a variety of cancer cells [36,37,38,39,40,41]. In addition, though some studies have discovered the role of NR2F2 in early embryonic development and cardiovascular formation [42,43,44], It is of great interest to study the role of NR2F2 in the umbilical cord both in mouse and human studies. Thus, investigating the developmental process and physiological functions of the umbilical cord using NR2F2-knockout mice could provide novel insights into the functions of NR2F2 in vivo. Nonetheless, our research proves that NR2F2 plays an important role in mesenchymal stem cells, which can not only affect cell proliferation but also have an impact on immune regulation. Therefore, clarifying the molecular mechanism by which NR2F2 regulates the growth of WJ-MSCs and exerts anti-cancer effects is beneficial to deepen the understanding of WJ-MSCs and provide new ideas for cancer treatment.

## 5. Conclusions

In conclusion, our study established that NR2F2 not only plays an important role in the gene regulatory network involved in the major biological processes of WJ-MSCs but may also have effects in immune modulation.

## Figures and Tables

**Table 1:** Primer sequences of target genes in qPCR.

Gene	Primer Sequences (5′-3′)
*β-actin* (Human)	F:CAGGGCGTGATGGTGGGCAT R:GATGCCGTGCTCGATGGGGT
*NR2F2* (Human)	F:GTTTGTGTTGAATGCGGCGCAGTG R:TGGGCTACATCAGAGAGACCACAGGCATC
*IL6* (Human)	F:AGACAGCCACTCACCTCTTCAG R:TTCTGCCAGTGCCTCTTTGCTG
*IL8* (Human)	F:GAGAGTGATTGAGAGTGGACCAC R:CACAACCCTCTGCACCCAGTTT
*ALDH1A1* (Human)	F:GGAATACCGTGGTTGTCAAGCC R:CCAGGGACAATGTTTACCACGC
*BMP6* (Human)	F:CAGCCTGCAGGAAGCATGAG R:CAAAGTAAAGAACCGAGATG
*DSG2* (Human)	F:CTCTTTCCGAGCTAGTGAGGCA R:CTGAAGTGACGGAGTCCACAGA
*CD200* (Human)	F:GAAGGTCTCAGGAACAGCTTGC R:GCAGTCGCAGAGCAAGTGATGT
*MMP1* (Human)	F:CTCTGGAGTAATGTCACACCTCT R:TGTTGGTCCACCTTTCATCTTC
*CDK4* (Human)	F:CCGACCAGTTGGGCAAAAT R:GATACATCTCGAGGCCAGTCATC
*CDK6* (Human)	F:GGTACAGAGCACCCGAAGTCTT R:AGCCAACACTCCAGAGATCCA
*CCND1* (Human)	F:GCATGTTCGTGGCCTCTAAGA R:CGGTGTAGATGCACAGCTTCTC
*NFKB* (Human)	F:TGTCCAGCTTCGGAGGAAAT R:TACCACCGCCGAAACTATCC

## Data Availability

Not applicable.
